# Accessibility and Historical Change: An Emergent Cluster Led *Uncles and Aunts* to Become *Aunts and Uncles*

**DOI:** 10.3389/fpsyg.2021.662884

**Published:** 2021-05-26

**Authors:** Adele E. Goldberg, Crystal Lee

**Affiliations:** Psychology Department, Princeton University, Princeton, NJ, United States

**Keywords:** binomials, historical change, American English, accessibility, cluster or neighborhood effect, emergent generalization

## Abstract

There are times when a curiously odd relic of language presents us with a thread, which when pulled, reveals deep and general facts about human language. This paper unspools such a case. Prior to 1930, English speakers uniformly preferred male-before-female word order in conjoined nouns such as *uncles and aunts; nephews and nieces; men and women*. Since then, at least a half dozen items have systematically reversed their preferred order (e.g., *aunts and uncles, nieces and nephews*) while others have not (*men and women*). We review evidence that the unusual reversals began with *mother and dad(dy)* and spread to semantically and morphologically related binomials over a period of decades. The present work proposes that three aspects of cognitive accessibility combine to quantify the probability of A&B order: (1) the relative accessibility of the A&B terms individually, (2) competition from B&A order, and critically, (3) cluster strength (i.e., similarity to related A'&B' cases). The emergent cluster of female-first binomials highlights the influence of semantic neighborhoods in memory retrieval. We suggest that cognitive accessibility can be used to predict the word order of both familiar and novel binomials generally, as well as the diachronic change focused on here.

## Introduction

Before the 1930s, English speakers systematically preferred the following word orders when using pairs of common nouns referring to male and female entities: *uncle and aunt, nephew and niece, pa and ma, grandpa and grandma, father and mother, grandfathers and grandmothers*. These orderings all reflected a preference to produce the male term first, a preference which remains generally operative today (Levy, 2005; Wright et al., 2005; Lohmann and Takada, 2014; Iliev and Smirnova, 2016; Tachihara and Goldberg, 2021). Yet by 2010, English speakers came to prefer the reversed order in each of the phrases just mentioned (e.g., *aunts and uncles, nieces and nephews, grandma and grandpa)*.

**Figure 1:**
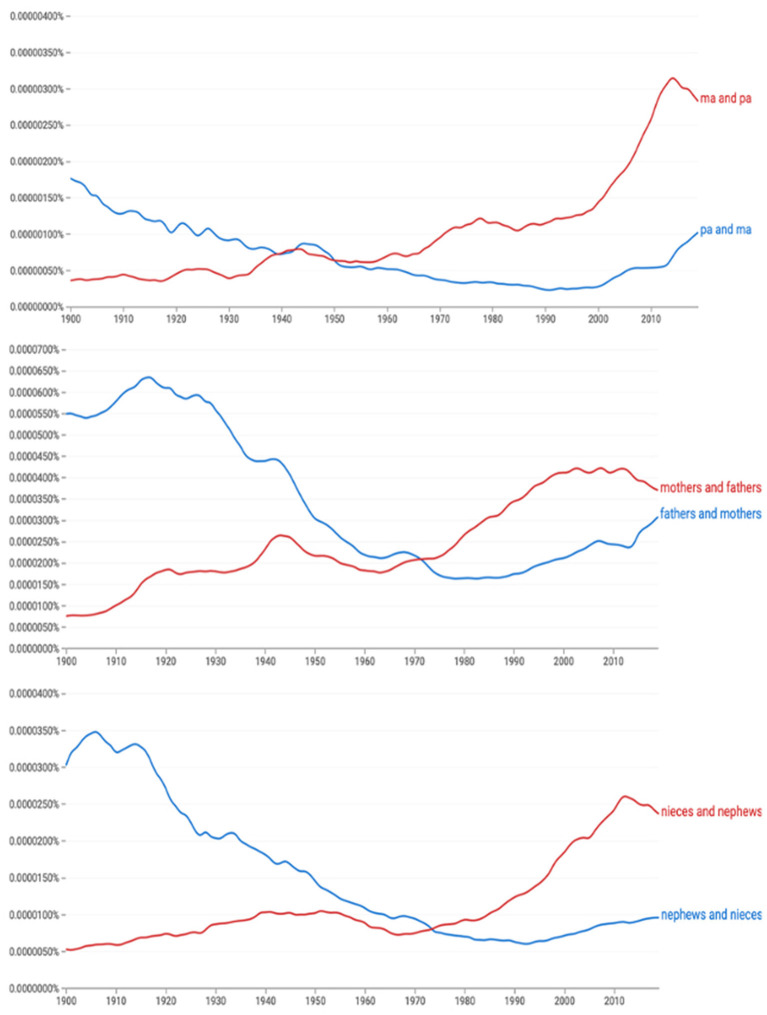
The historical shifts in preferred order from *pa* and *ma* to *ma* and *pa* **(top)**, from *fathers* and *mothers* to *mothers* and *fathers* **(middle)**, and from *nephews* and *nieces* to *nieces* and *nephews* **(bottom)** in Google Books N-gram (Michel et al., 2011). The x-axes represent time 1900–2019 and the y-axes represents the relative percentages of each order. Female-first order is represented in red and male-first in blue.

Figure 1 provides three examples of the unusual reversal of preferred word order. The x-axes in each panel represent decades from 1900 to 2019 and the y-axis represents the relative percentages of each order in the Google N-gram online corpus, containing 500 billion words (Michel et al., 2011). Female-first order is represented in red, and male-first, in blue. “Pa and ma” shows the shift to *ma and pa* order as early as 1950 (top); “fathers and mothers” displays a reversed preference of *mothers and fathers* by roughly 1970 (middle), and “nephews and nieces” shows the preference reversal by the mid 1970s (bottom).

**Figure 2:**
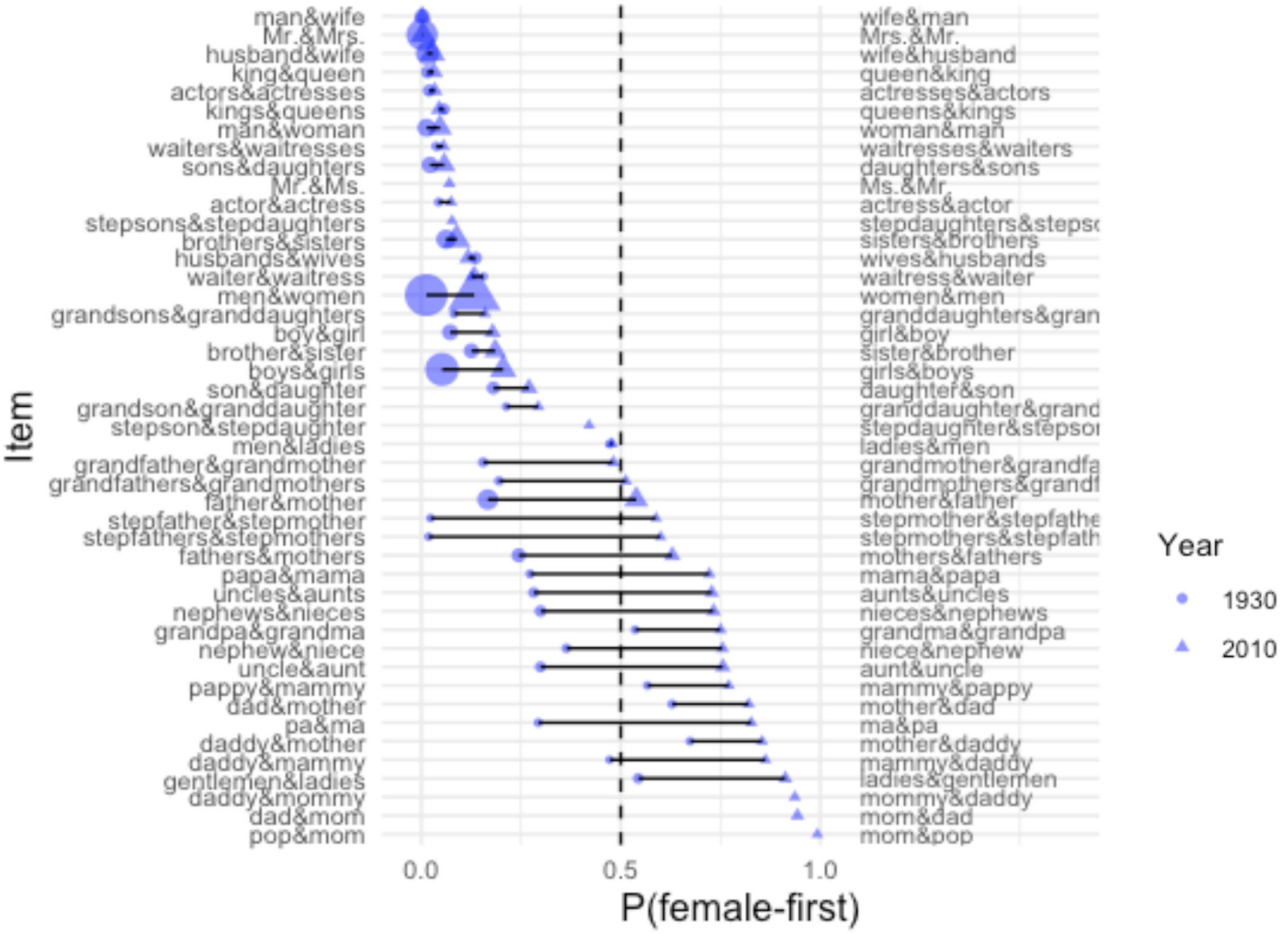
The probability of female-first order for 45 gendered binomials at two time points: 1930 (circles) and 2010 (triangles), based on data from Google Books N-grams. The size of the endpoints represents the frequency of the binomial (in either order). Time points (and lines) are omitted for items below a threshold frequency of 0.05 per million words.

Figure 2 represents the difference in probability of female-first order at two time points: 1930 and 2010, for each of the 45 items included in the current analysis. The length of the lines represents the extent of the shift for each item. The size of the endpoints represents the relative token frequency at the two time points (regardless of order). As is clear from Figure 2, the word-order preference has not shifted equally in all items.

Here we ask, what caused the word order shift and why have some gendered binomials shifted more than others? The question arises because the order of words in familiar phrases rarely changes (e.g., Bybee, 2002; Hopper and Traugott, 2003; Brinton and Traugott, 2005; Traugott and Trousdale, 2013). Accordingly, reversals in the preferred word order of familiar binomials are highly unusual; instead, people tend to reproduce binomial phrases in the same order they have witnessed them used (Malkiel, 1959; Cooper and Ross, 1975; Morgan and Levy, 2016). For instance, Mollin (2013) documented the ordering of more than 200 frequently occurring binomials across two centuries. Of those, 93% displayed a preference toward one order over another. She found only 1% of binomials reversed their preferred order, observing that *mother and father* was such a case, as we return to in the section, Social Influences.

Given the rarity of reversals in word order, it is striking that a dozen gendered binomials have come to reverse their preferred order, displaying a female-first preference today, despite a continuing bias in English toward male-first order in other cases. In what follows, we aim to explain and quantify *why* certain cases have reversed their preferred order, and why other cases have not undergone the reversal, at least not yet (e.g., *brothers and sisters*). In doing so, we discuss and relate several factors that are recognized to play a role in novel and familiar binomial order more generally. We then provide a quantitative analysis, based on a century of data culled from Google Books Ngrams.

Mollin (2013) offers a detailed diachronic study of binomial terms that is particularly relevant in the current context (see also Mollin, 2014; Kopaczyk and Sauer, 2017). While we note that reversals in preferred word order are rare, she emphasized that changes in degree of fixedness are common. Degree of fixedness refers to the ratio of tokens ordered one way over the number of tokens ordered in either way. Mollin (2013) argued that decreases in fixedness appear to pose a challenge to usage-based models of language because they represent a shift away from conventionality toward novelty. That is, usage-based models of language predict speakers' productions should reflect the statistical regularities they witness, so any systematic change requires explanation. In fact, many changes have been studied and explained through processes of reduction (Frishberg, 1975; Hopper and Traugott, 2003; Brinton and Traugott, 2005), reanalysis (Langacker, 1977; Eckardt, 2006), language contact (Thomason, 2003), or changes in meaning or emphasis (Traugott, 1988; Grieve-Smith, 2019). However, none of these standard explanations applies to the shift toward female-first order in gendered binomials: Female-first binomials are not reduced versions of male-first orderings; they are not analyzed differently than male-first orderings, as all are transparent conjunctions; and they are not a result of language contact, as none of the terms is borrowed. Mollin (2013) proposes that the change is due to the women's liberation movement in the 1970's, an intriguing idea we discuss in the following section. Ultimately, however, the timing and specifics of the change lead us to propose a more mechanistic account.

Much has been written about factors that make one word in a binomial more likely to be produced first. As discussed below, these factors include frequency, definiteness, and priming, as well as semantic animacy, relevance to the speaker, prototypicality, and concreteness (Cooper and Ross, 1975; Benor and Levy, 2006; Onishi et al., 2008; Lohmann and Takada, 2014; Morgan and Levy, 2016; Tachihara and Goldberg, 2021). As additionally reviewed below, a good deal of work has also demonstrated that prior experience with one order or the other predicts future uses (Cooper and Ross, 1975; Mollin, 2013; Morgan and Levy, 2015; Conklin and Carrol, 2020). Yet neither of these factors on its own predicts a *change* in word order, because the meanings of the terms has hardly changed, and previous male-first word order *failed* to persist across diachronic time.

We propose that the historical shift was precipitated by two independently motivated female-first binomials, namely *mother* and *daddy* and *mother* and *dad*. These cases initiated an emergent cluster of female-first binomials which began to slowly attract highly similar binomials, particularly binomials that were not themselves highly entrenched in the opposite, male-first order. The emergent cluster illustrates how new sub-regularities can arise in language.

We also suggest a unified account of binomial orderings based on the *cognitive accessibility* of the parts and the whole, where cognitive accessibility refers to the speed or ease with which concepts are retrieved from memory (Tulving and Pearlstone, 1966; see also Ferreira and Dell, 2000; MacDonald, 2013). Although “accessibility” is not often mentioned in work on historical change, nor even in work on binomial word order (except in Onishi et al., 2008; Lohmann and Takada, 2014; Tachihara and Goldberg, 2021), we argue that A&B order is predicted by the relative accessibility of the parts (A vs. B) and the degree of competition from the alternative order (B&A). In what follows, we describe how prior work on binomials can be interpreted straightforwardly in terms of accessibility, and importantly, we provide evidence for a third additional factor, *cluster strength*. The emergent cluster of related cases serves to motivate the historical shift in word order, and we argue, is also directly related to cognitive accessibility. We thus propose unifying these three factors affecting binomial order by observing that they jointly determine which order is more accessible from memory, as represented in (1):

(1) P(A&B) ~ Cognitive accessibility of the A term compared to the B term
− Competition from B&A
+ Cluster strength of binomials related to A&B (A'&B')

After discussing a possible role for social factors, we propose the catalyst for the historical change. We then quantify each of the relevant factors (section Cognitive Accessibility), and explain how each is related to accessibility. This allows us to test a multiple linear regression model that combines the proposed factors.

## Social Influences

**Figure 3:**
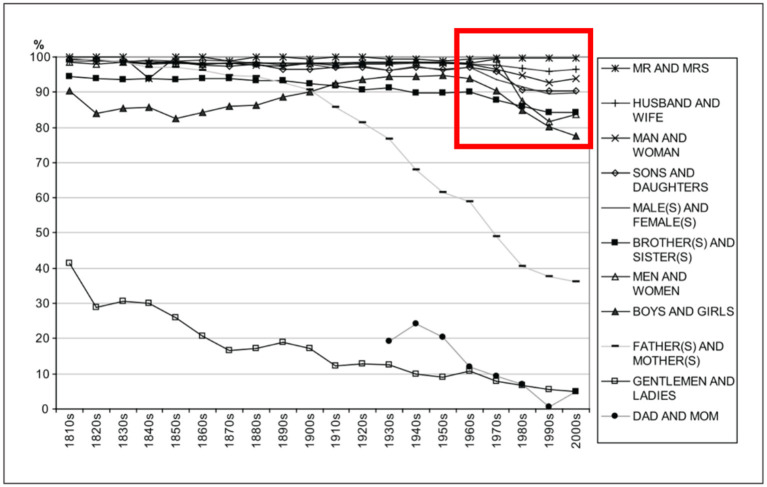
Examples of gendered binomials which show an increasing trend away from male-first order during the 1970s as indicated by the red box (added). From Mollin (2013, Figure 9).

Mollin (2014) thoroughly documents and reviews the historical ordering of over 200 of the highest-frequency binomial expressions in English between 1800 and 2000. In Mollin (2013), she observes a decrease in fixedness away from male-first ordering among several high-frequency gendered binomials in the 1970s, as depicted in the red box in Figure 3 (Mollin's, 2013, Figure 9, box added). For example, while “boys and girls” overwhelmingly preferred male-first order before 1970, the percentage of male-first order (*boys and girls*) was reduced from nearly 100% to closer to 80% by the 2000s.

Because the decrease in fixedness of these cases appears to begin in the 1970s, Mollin suggests that the shift was caused by the changing cultural roles for women in American society during the wave of advocacy for gender equality in that period. We take this to suggest that the change was due to a difference in construal: as females began to be viewed as more and more equal to males in terms of perceived power, agency, and importance, the semantic motivation for male-first order was weakened. To presage the current results, we do find a general decrease in male-first order over the past century. But the timing of the shift among gendered binomials was gradual, with reversals of preferred order observable across decades. As Mollin (2013) acknowledges, the unfreezing of *mother and father* began well before the 1970s, and *mom and dad* has always preferred female-first order (Figure 3). To address this, she suggests that terms referring to parents may require a special explanation:

“If we consider the changes plotted in Figure [3**]** …in more detail, it is interesting that the binomials in which the tendency to name the woman first … are those referring to the *parental* roles of men and women [emphasis added]: contrary to usage in the nineteenth century, in which *father(s) and mother(s)* was strongly preferred, we have witnessed an unfreezing trend to the point of reversibility, with a mild preference today to name mothers first. One may speculate that this is because the mother's typically more central role in child raising is now seen to be more important than the traditionally larger familial authority of the father. … All parental binomials now prefer the female element to come first, especially *Mom and Dad*, which is almost frozen” (Mollin, 2013, p. 196).

We argue that a trend toward more gender-equality cannot explain the full pattern of results. Because Mollin (2013) considered the most frequent binomials, her dataset did not include the full set of gendered binomials considered here. For instance, *nephews and nieces, uncles and aunts*, and *grandfather and grandmother* were not included, because they are not among the most frequent binomials. Yet the shift away from male-first order also began in these cases even though the societal roles played by nephews vs. nieces, uncles vs. aunts, or grandmothers vs. grandfathers have not changed dramatically, or at least not in ways that garnered much public discussion or awareness.

Critically, the male-before-female preference has been *reversed* in more than a half dozen gendered binomials (recall Figure 2). This is challenging to reconcile with the fact that English speakers continue to display a male-first bias elsewhere. For instance, Wright et al. (2005) reports a male-first bias (in addition to a shorter-first bias) when participants were asked to produce first names that were stereotypically male or female. Tachihara and Goldberg (2021) likewise reports a male-first bias when people name familiar couples, over and above effects of length and reported feelings of closeness. Finally, Lohmann and Takada (2014) found a male-first bias in modern corpus data. Thus, a general (potentially weakened) male-first bias remains evident in English outside of the cases that are the focus of the current analysis^1^.

We do not mean to dismiss the importance of societal stereotypes. Hegarty et al. (2011) demonstrated that the male-first preference itself arises from a stereotypical difference in perceived power or masculinity. In particular, they asked participants to name fictional gay couples, in which one member of each couple was described as having a trait that was considered stereotypically more masculine. Participants were told, for instance, that one member of each couple earned more money or was physically larger. Results showed that participants tended to name the person assigned the stereotypically masculine property before the other member of the couple. Since both members of each couple were men or both were women, perceived dominance and not gender per se was responsible for this bias in word order. Presumably the cultural stereotype that assigns males more dominance or power underlies the continuing male-first bias (see also Benor and Levy, 2006). A general trend toward greater perceived gender-equality cannot fully account for the ordering reversal among the cluster of cases focused on here given that an overall male-first bias in binomials remains evident. Yet, it is possible (and we are optimistic) that the difference in perceived dominance between males and females has lessened over the past century, and it is reasonable to assume that a perceived difference in dominance may vary across different binomials (boys may be viewed more equitably compared to girls than kings are in comparison to queens, for example). We return to this point in the Discussion, observing that such changes would be a welcome addition to the current account.

## Ground Zero for Female-First Binomials: *Mother and Dad(DY)*

If the shift is not due to increasing gender equality in the 1970s, when and how did it begin? In what follows we offer a mechanistic account based on cognitive accessibility. The analysis is based on data collected as follows.

In order to determine the frequencies of individual terms and binomial phrases as units, we analyzed the largest corpus available, Google Books N-grams, which includes roughly 500 billion words (Michel et al., 2011)^2^. The same trend toward female-first order among a cluster of binomials is also evident in COHA, the corpus of historical American English (Davies, 2010). However, the frequency estimates for smaller corpora are less reliable, so we use the larger corpus, even though it is not ideal (Pechenick et al., 2015). For example, corpus size is not stable across time points in Google Books N-gram. To address this, we use the percentages provided by Google N-grams at each decade rather than raw frequencies. To obtain frequency information that was comparable, we multiplied percentages by one million, a very conservative estimate of corpus sizes. We then converted frequencies to a log scale, since log values are recognized to predict accessibility (Carroll, 1967; Baayen, 2002; Balota et al., 2004). The data was comprised of the 45 binomials listed in Figure 2 (and in Appendix). For each binomial, we sampled frequencies in both orders, for the first year of each of 10 decades: 1920, 1930, 1940…2010, and in 2019, the last year available.

**Figure 4:**
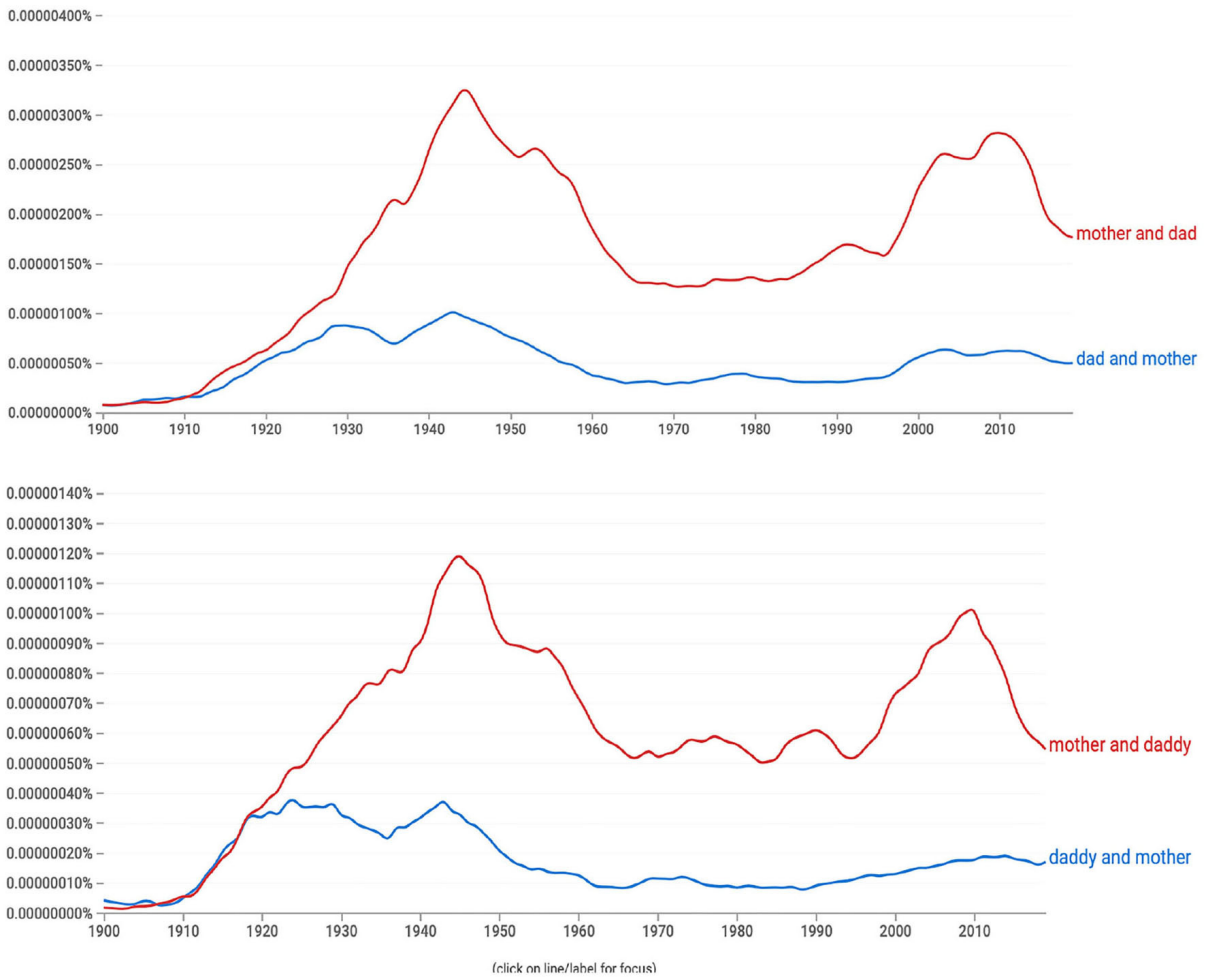
**(Top)** Mother and dad was more common than dad and mother (1900–2019), **(Bottom)** Mother and daddy was more common than daddy and mother since (1900–2019).

We propose that the subregularity of female-first binomials evident today was catalyzed by the binomials, *mother and daddy* and *mother and dad*. Both displayed a preference for female-first order as early as any preference is detectable, circa 1920 as shown in Figure 4.

Although uncommon today, both binomials were more commonly used than *mom and dad* before 1950 (Google Books Ngram) (Emily Morgan, personal communication, 2/16/21)^3^. Why did these cases show the preference for female-first order, flouting the robust male-first bias at that time?

Recall that the male-first preference is itself part of a more general tendency to position the label of a referent perceived to be more stereotypically masculine, powerful or important first (Benor and Levy, 2006; Hegarty et al., 2011). This raises the possibility that the female-first order of *mother and dad(dy)* was motivated by an interpretation of *mother* as the parent who was more powerful or in control than *dad (*or *daddy*). Indeed, *mother and daddy* sounds odd today, since *mother* is a formal term, used primary by adults, while *daddy* is informal, affectionate, and typically used by children. Yet the earliest uses of these phrases offer little indication that the female parent (*mother*) was construed as more in control or more important than the male parent (*daddy)*. Consider a typical early example, in (2) (boldface added). The character, Anita, speaks directly to both parents, but asks permission specifically from her father, indicating that he has more authority; moreover, in the same conversation, Anita refers to her parents as *father and mother*, a male-first binomial^4^:

(2) “I have been to see Mrs. Lawrence,” said Anita, “and she asked me if I would write a letter for her. She didn't, of course, tell me not to say anything about it to you, **mother and daddy**, but I would rather not tell you to whom the letter is to be written. You must trust me, my own dear daddy. It is a very simple letter, just to say that Lawrence has disappeared and Mrs. Lawrence and the little boy are in kind hands.” “Of course we trust you,” answered Colonel Fortescue, smiling. “You are a very trusty person, Anita.” “Like my **father and mother**,” answered Anita (COHA, 1916, Betty at Fort Blizzard by Seawell, 1916).

The binomial terms in *mother and daddy* are asymmetric in that *daddy* was and remains an affectionate appellation, especially in comparison to the more formal relational term, *mother*. This may imply a closer relationship between the child and male parent. However, to the extent that this is true, it predicts that *daddy* should have been ordered before *mother*, not the other way around. That is, when speakers refer to familiar couples, they tend to order the individual they feel closer to before the other member of the pair (in both English and Japanese, despite the languages' different overall word orders; Tachihara and Goldberg, 2021; see also Lohmann and Takada, 2014). To summarize, insofar as *daddy* is a term of endearment and *mother* is more formal, the reason speakers in the 1920s violated the general bias toward male-first order in the case of *mother and daddy* and *mother and dad* is not likely due to the semantic and pragmatic properties of the individual terms.

**Figure 5:**
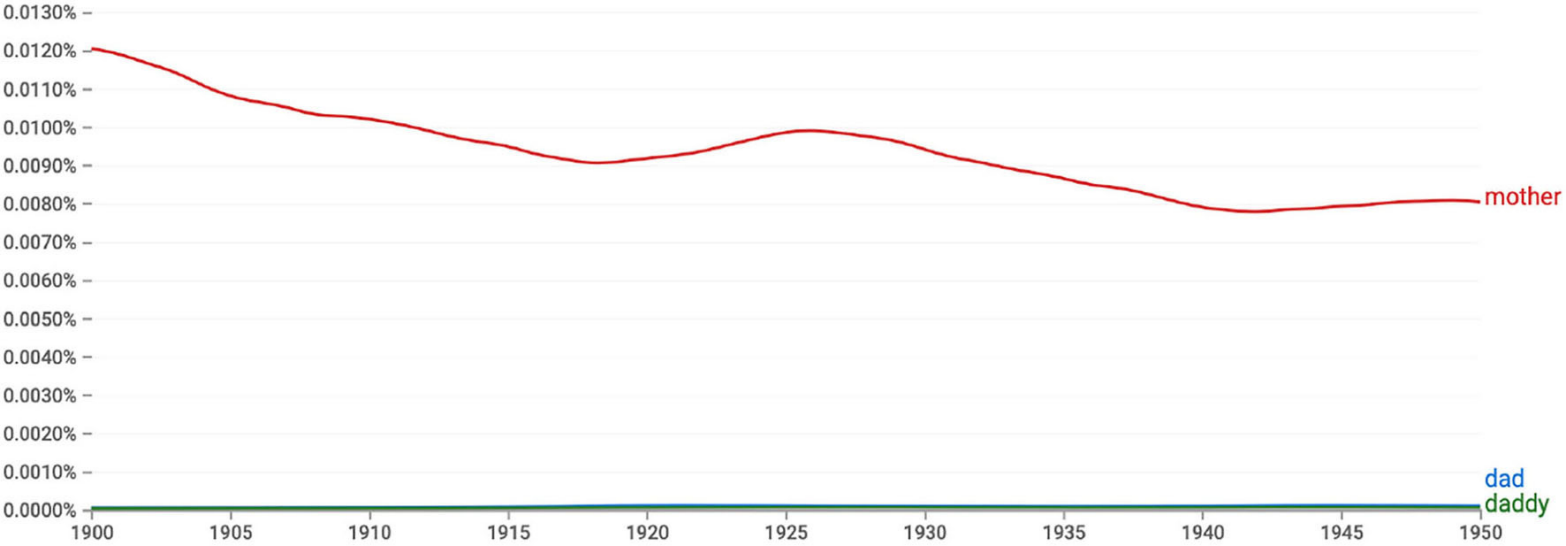
The frequencies of individual terms: *mother* was two orders of magnitude more frequent than *daddy* or *dad* during the period when *mother* and *daddy* and *mother* and *dad* emerged (see this figure, 1900–1950).

A more compelling explanation for the order of *mother and dad(dy*) is that *mother* was roughly 100 times more frequent than *daddy* or *dad*. Figure 5 illustrates this massive difference in frequency: the relative frequencies of *daddy* and *dad* are so low that they are hard to see in the figure. We suggest that the dramatic difference in frequency resulted in *mother* being more cognitively accessible than *daddy* or *dad*, making it likely that the word *mother* would be retrieved from memory more quickly than either *daddy* or *dad*. This then explains why *mother* was originally ordered before *daddy*, even though nearly all other binomials at the time preferred male-first order.

The analysis below suggests that the appearance of *mother and daddy* and *mother and dad* led to a cascading effect across semantically and morphologically similar cases, resulting in the cluster of female-first binomials that exists today. More generally, as we detail, the root cause of *mother and daddy*'s order *and* the gradual shift among many other gendered binomials *and* the ordering of conventional binomials *and* the ordering of novel binomials is: cognitive accessibility. We review three relevant aspects of accessibility immediately below.

## Cognitive Accessibility

As noted in the introduction, *cognitive accessibility* refers to the speed and accuracy of recalling a memory trace, regardless of whether the memory represents a semantic concept, an episodic event, a word, or a phrase. The intended message is by far the most important factor determining which words and constructions are accessed when we produce language in natural contexts (e.g., Tulving and Pearlstone, 1966). That is, we generally use words and constructions that are well-suited to the messages we want to convey, recalling, and combining them from long-term memory appropriately. While errors can and do occur, most utterances are at least “good-enough” to avoid catastrophic misunderstandings because speakers successfully access words and constructions that are semantically appropriate. Given this, we assume that if A&B and B&A were associated with different meanings, the intended message would predict which order is expressed. Yet in the cases of gendered binomials focused on here, *aunts and uncles* conveys the same content as *uncles and aunts*, aside from possible differences in emphasis, which we return to in the Discussion. We therefore put aside any differences in the overall semantic construal of one order over the other when considering the relative accessibility of male-first vs. female-first order.

In wain how familiar and proposed influences on binomial order are related to aspects of cognitive accessibility. The proposed factors combine, we suggest, to predict the order of gendered binomials across the century, including the shift in preferred order of particular interest here. In this section we explain how the probability of producing the order, A&B, rather than B&A, is predicted by the factors repeated in (3). We explain how each factor is quantified in turn below.

(3) P(A&B) ~ Relative accessibility of A, B terms individually
− Competition from B&A
+ Cluster strength of cases related to A&B (A'&B').

### Calculating the Relative Accessibility of A, B Terms Individually

Much has been written about the factors that influence which term in a binomial is expressed first^5^. Benor and Levy (2006) assessed the role of 20 different factors, while Morgan and Levy (2016, p. 389) usefully narrowed the relevant influences to those in (4):

(4) (i) General before specific: (e.g., *boards and 2x4s*)
(ii) Perceptual markedness (animates first, self before others, concrete before abstract)
(iii) Powerful first: culturally prioritized (e.g., male first, alcohol first)
(iv) Iconicity: order reflects temporal order of events (e.g., *sit and stay*; *achieved and maintained)*
(v) Higher frequency first
(vi) Shorter length first.

Importantly, experimental work investigating the recall or accessibility of individual words has found parallel influences, related to semantics (i–iv), frequency (v), and length (vi) (Tulving and Pearlstone, 1966; McDonald et al., 1993; Müeller, 1997; Wright and Hay, 2002; Indefrey and Levelt, 2004). We address each in turn. Semantics is recognized to influence the relative accessibility of different words in experimental contexts in which the overall message to be conveyed is held constant or is not relevant to the task. For example, other things being equal, words that label animate entities, concrete entities, or words with an emotional valence tend to be accessed before inanimate, abstract, or neutral words, respectively (Balota et al., 1991). In the case of the gendered binomials under consideration, the content of the terms is well-matched along most semantic dimensions: generality and iconicity are not relevant to gendered binomials, and both terms refer to agentive humans, who fulfill largely symmetric roles. The primary difference in meaning between the two terms is systematic and simple: gender. As already discussed, entities that are perceived to be more powerful or important tend to be named first and we assume that males tend to be named first because they are stereotypically perceived to be more powerful. While little work has directly tested whether words referring to males are generally easier to access than words referring to females, there is suggestive evidence that words that refer to stronger or more “potent” entities *are* more accessible than words that refer to weaker or less “potent” entities (Osgood, 1969; Wurm et al., 2004). Relatedly, more powerful entities are more likely to be encoded as agents (Frenzel et al., 2015). We capture the idea that terms referring to entities perceived to be more dominant are more accessible by simply adding a fixed numerical weight (1.0) to all male terms in the calculation of their accessibility. It is possible that this value has changed over the past century, as women have become more independent and powerful in society (recall Social Influences). We also recognize that what we treat as a fixed value added for dominance should perhaps vary depending on the binomial involved (e.g., perhaps the difference in perceived dominance between the terms *Mr*. and *Ms.*, should be less than that between *Mr*. and *Mrs*., for instance). We return to this point when we discuss the results of the model. The current analysis is conservative on this point, however, as we leave the value of the dominance constant fixed at 1.0.

Frequency is also known to play an important role in accessibility during recall generally, and in binomial order, specifically. Words that have been encountered more frequently tend to be accessed faster (e.g., Forster and Davis, 1984). Every psycholinguistic study takes frequency into account when predicting the speed and accuracy of retrieving words from memory, regardless of whether the task is lexical decision (“is this a word or not?”), comprehension, picture naming, or repetition. When words are presented without semantic context, frequency is an excellent predictor of the N400, a component in ERP analyses implicated in lexical access (King and Kutas, 1995). As expected then, the more frequent term in a binomial tends to occur first. For instance, Fenk-Oczlon (1989) was the first to recognize the importance of this factor and found the higher frequency word to be ordered first in 84% of the 400 binomials she examined. Benor and Levy (2006) likewise found the relative frequency of individual terms to be predictive, although less strongly than Fenk-Oczlon had (roughly 68% of cases positioned the more frequent word first). The influence of the relative frequencies of the individual terms is a straightforward effect of accessibility. Since the frequencies of individual terms can vary across decades, the current analysis determines (log) frequencies at each decade.

Finally, the relative length or complexity of words or phrases is also known to affect cognitive accessibility in general, and binomial order in particular: holding frequency constant, shorter simpler words, and phrases are easier or faster to retrieve from memory than longer words or phrases (Baddeley et al., 1975; McDonald et al., 1993; Müeller, 1997; Wright and Hay, 2002; Levelt and Sedee, 2004; Benor and Levy, 2006). We therefore include length (in syllables) in the determination of the relative accessibility of each male and female term, by subtracting the number of syllables of each term in the calculation of its accessibility.

It would be possible (and some might say preferable) to treat semantics, frequency, and length as distinct factors. However, we combine them here to reduce the number of parameters and to emphasize our belief that they combine to predict relative accessibility. To summarize, as represented in (5), we calculate the relative accessibility of the female term relative to the male term by subtracting the cognitive accessibility of the male term from the female term:

(5) Relative cognitive accessibility of F vs. M terms:
[*logfreq(Fem) – #syllables*]_accF_ – [*logfreq(Masc) – #syllables* + 1.0]_accM_

By subtracting the accessibility of one term from the other, we capture the fact it is the relative accessibility of the terms that matters since one or the other of the two terms must be expressed before the other.

Other effects known to be relevant to binomial ordering and accessibility more generally include definiteness and priming. That is, if one term is more identifiable or more primed in the discourse context, the likelihood of it being pronounced first increases (Morgan, 2016, chapter 3; Benor and Levy, 2006). We do not include these factors in the current analysis only because it was not feasible to hand-code the passages surrounding binomials in Google Books Ngrams. Fortunately, there is no reason to assume that one or the other term should be systematically more topical or primed in contexts in which a binomial is used, beyond asymmetries in the frequencies of the individual terms, which are taken into account in the formula in (5). As we return to in the Discussion, the relevance of priming and definiteness lends support to the current claim that accessibility provides a unifying construct.

It turns out that the terms for females are rarely more frequent than the male term in our binomials, and this together with the positive constant for entities perceived to be more powerful or masculine, the female terms in nearly all of the gendered binomials in the data set are, in absolute terms, less accessible than the male terms. The two exceptions, as already discussed, are *mother and daddy* and *mother and dad*. Thus, the factor in (5)—the relative accessibility of the individual terms—cannot on its own predict a shift from male-first to female-first order.

### Competition From B&A

The usage-based constructionist approach which we adopt treats words, familiar phrases, and grammatical constructions as learned pairings of form and function, represented in a complex web that comprises our knowledge of language (e.g., Bybee, 2010; Goldberg, 2013; Kapatsinski, 2018). As expected, the frequencies of phrases influence how quickly and accurately they are accessed as units and this has been found to be the case in children and adults (Bannard and Matthews, 2008; Arnon and Snider, 2010; Ambridge et al., 2015; Arnon et al., 2017; Christiansen and Arnon, 2017). By the same token, the frequencies of each binomial as a unit influences its preferred word order as well. For instance, in a large self-paced reading-time study, Morgan and Levy (2016) report that prior experience with A&B binomials results in faster reading times for the A&B order and slower reaction times for B&A order, with a stronger effect as the frequency of A&B increases. As they emphasize, the influence of the frequency of the binomial as a unit supports the usage-based claim that multi-word units are retained in memory. Conklin and Carrol (2020) found converging results with newly-introduced binomials; reading times decreased as exposure increased, while reading time to the reversed order decreased as exposure increased. Similarly, the most frequently occurring cases tend to be more fixed in their order than less frequent cases (Cooper and Ross, 1975; Gustafsson, 1976; Mollin, 2013; Morgan and Levy, 2015).

Accessibility is recognized to be negatively affected by interference or competition (e.g., Underwood, 1957). In fact, memory researchers have found that if a memory is partially activated but repeatedly loses in a competition with another memory, the former becomes more difficult to subsequently access (Anderson et al., 1994). We can therefore expect that the more frequent or entrenched a binomial is in one order, the less likely it will be to reverse its order. That is, insofar as the meaning of gendered binomials is unaffected by order, witnessing one order is tantamount to not witnessing the other order. We therefore describe the influence of the frequency of the binomial as a unit in terms of competition: greater familiarity with one order leads to greater interference from that order during the production or comprehension of the other order. This can be described as statistical preemption: repeatedly witnessing a particular gendered binomial (“A&B”) in one order *statistically preempts* the use of the other order with the same intended meaning (e.g., Boyd and Goldberg, 2011; Goldberg, 2011, 2019; Perek and Goldberg, 2017). This entails that binomials appearing more frequently in the male-first order should compete more strongly against the female-first order being used. The strength of competition for female-first order is defined here as the log frequency of the male-first order, which (again calculated for each decade) is defined in (6).

(6) Competition: *logFreq*(*M&F*)

The inclusion of this term could raise concerns about circularity if low frequency of M&F order entails high probability of female-first order. However, this is not the case. The frequency of the male-first order can be low even if the probability of female-first order is also low, as is the case in many low-frequency binomials in our dataset (e.g., *actor and actress, Mr. and Ms., waiter and waitress*). We expect competition from male-first order to be a negative predictor of female-first order.

### Cluster Strength

The final factor relevant to accessibility is *cluster strength*, which is key to motivating the historical shift toward an increased probability of female-first order. Cluster strength is often discussed under the notion of “neighborhood density.” We prefer the term *cluster strength* for three reasons. First, neighborhood effects are typically discussed in terms of phonological or orthographic neighbors rather than semantic or morphological neighbors, while the latter are relevant here. Secondly, work on neighborhood density has often focused on an inhibitory effect, which occurs when neighbors require an incongruent response. For instance, in lexical decision tasks (i.e., “is this a word?”), responses to *non*-word strings are slowed by real-word neighbors. That is, it takes longer to recognize that *strink* is not a word (many neighbors, e.g., *stink, string)* than it takes to recognize that *ngilm* is not a word (Forster and Shen, 1996; Baayen et al., 2006; Hendrix and Sun, 2020). What is relevant for our purposes is that cluster strength is recognized to be faciliatory in recall tasks, and when neighbors allow a congruent response (Roodenrys et al., 2002; Derraugh et al., 2017). For instance, Pecher et al. (2005) required participants to determine whether a word referred to an animate entity or not; they found that reaction times to animate words (e.g., *cat*) were faster when the word had more animate neighbors (e.g., *rat, bat*). Finally, we prefer cluster strength because the relevant factor is relational: cluster strength is not dependent on the size or density of a cluster of cases on its own, but is instead dependent on the relationship between an item and a cluster (see also Suttle and Goldberg, 2011; Goldberg, 2019).

To appreciate the relevance of cluster strength, it is important to recognize that memories are represented in a distributed, associative network. This is clear from decades of work on priming (e.g., Tulving and Schacter, 1990; Hoey, 2005). For example, thinking about the ocean often leads to thoughts of the beach; relatedly, the word *ocean* primes the word *beach*, making the latter more accessible and easier to recall. Similarly, we can expect the phrase *aunts and uncles* to prime *nieces and nephews*. We can also expect *mother and daddy* to prime *ma and pa*. Insofar as a cluster of similar cases is primed when a speaker plans to produce a binomial, it will facilitate the production of a congruent word-order; In the current case, female-first word order.

Thus, key to the current proposal is the idea that an emergent cluster of female-first binomials has attracted other binomials, to the extent that other binomials are semantically and morphologically similar to instances in the cluster. To avoid circularity, we calculate cluster strength of each binomial at decade, *n*, on the basis of other binomials which had already shown a female-first bias in the previous decade, *n*−1. Cluster strength is the factor of most interest to us, as it is most relevant to the diachronic change. That is, the cluster of female-first binomials has gained strength over time as more gendered binomials assimilated into the cluster. To be treated as part of a cluster at each decade for the purposes of calculating similarity between binomials, we imposed two criteria. The binomial needed to have a preference, during the *prior* decade, for female-first order, however slight [P(F&M) > 0.50], and the binomial needed to occur with non-negligible frequency. Frequency was considered non-negligible if it was predicted to occur at least once in a corpus of one million words.

We considered the similarity, at each decade, between each binomial and each instance of the female-first cluster during the prior decade. While similarity is context-sensitive and can be challenging to calculate, the current focus on gendered binomials makes it possible to quantify it straightforwardly. We calculated morphological similarity and semantic similarity between any pair of binomials on the basis of 0–2 point scales as follows:

Semantic similarity between gendered binomials (F_1_, M_1_) and (F_a_, M_a_)

0.25: both are gendered binomials

1: both label relatives as part of a family tree: e.g., (*ma, pa)* and *(nieces, nephews*)
2: both label the same semantic relationship: e.g., (*ma, pa) (mother, father*)

Morphological similarity between (F_1_, M_1_) and (F_a_, M_a_)

0: no shared morphology: e.g., (*nieces, nephews)* and *(aunts, uncles*)
1: some shared morphology: e.g., (*ma, pa), (grandma, grandpa)*
2: one term is identical; or binomials differ only in plurality: *e.g., (mother, father), (mothers, fathers*)

Then, to determine cluster strength for each binomial at each decade, we additively compared each binomial with each unique binomial in the cluster in the prior decade^6^. Thus, the cluster strength between a binomial (F&M) and instances of the female-first cluster in the prior decade {F_i_ & M_i_, for all *i*} was determined as follows:

$$\begin{array}{l} \left(7\right)\overset{k}{\underset{i\;=\;1}{\sum }}[Sem\text{\_}sim\left({F\&M}_{decade\left(n\right)}\right),\;{F_{i}\&M_{i}}_{decade\left(n-1\right)}\\ \;+Morph\text{\_}sim\left({F\&M}_{decade\left(n\right)}\right),\;{F_{i}\&M_{i}}_{decade\left(n-1\right)}] \end{array}$$

Since our goal is to predict the proportion of female-first orderings, the cluster strength of other female-first orderings is predicted to be faciliatory.

**Figure 6:**
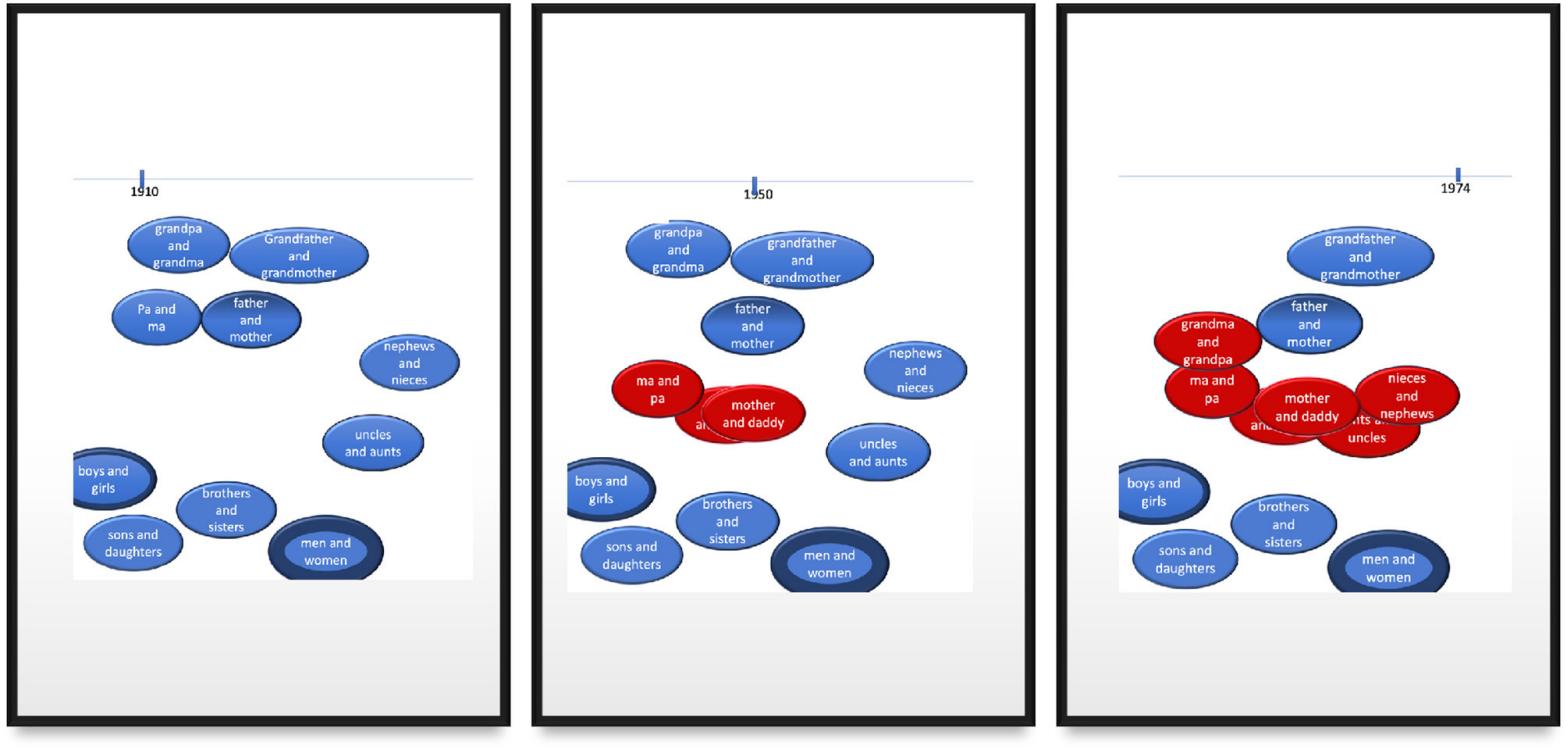
The three panels capture simplified snapshots of historical change for a handful of representative items. Blue circles represent a bias toward male-first order, while red circles represent a bias for female-first order. The thickness of the circles indicates their relative token frequencies (in either order).

Before we introduce the mixed model, it might be useful to consider oversimplified snapshots of a representative handful of cases depicted in Figure 6 at three time points (1910, 1950, 1974). The blue circles represent a >50% bias toward male-first order, while red circles represent a (changed) preference for female-first order. The thickness of each circle indicates that binomial's relative token frequency (in either order).

The left panel of Figure 6 captures the initial, virtually uniform bias toward male-first order that had existed in 1910 and before. The middle panel shows the innovative *mother and dad(dy)* along with *ma and pa*, which reversed its preference relatively early, due, we hypothesize, to a combination of its low frequency and high similarity to *mother and dad(dy*). The right panel is the preferred orders for the binomials by mid-1970; *aunt and uncle* and *nephew and nieces* had both shifted their preferences. Notably *father and mother* still preferred male-first order, presumably due to its relatively high frequency (= strong entrenchment) in male-first order, despite its high similarity to then-existing female-first cases (= high cluster strength). Closer to the bottom of each panel, we find *sons and daughters* which has not reversed its preference, despite labeling part of a family tree (= reasonably high cluster strength) and relatively low frequency; its failure to shift may be due to the fact that *daughters* is markedly less accessible than *sons* (it is less frequent *and* longer). Because such just-so stories might be told for a multitude of patterns, we aim to quantify the influence of each variable in the analysis below.

## Analysis and Results

We analyze the combination of factors defined as in (8) and their interactions, with the formula in (9). We registered an analysis at As.Predicted.org (https://aspredicted.org/xx635.pdf), but only after data for the 14 items had been collected so we do not claim it is preregistered^7^. Data and analyses are available at the Open Science Framework (https://osf.io/v6r53/).

(8) Proposed factors for the probability of female-first order (P[F&M])
Relative accessibility of F & M:
  $$\begin{array}{l} \beta_{1\;}[\left(logFreq\left(Fem\right)-\#syll\left(Fem\right)\right)\\ \;-\left(logFreq\left(Masc\right)-\;\#syll\left(Masc\right)+1\right)] \end{array}$$
Competition:
  $$\begin{array}{l} \beta_{2}\left[logFreq\left(M\;\&F\;\right)\right] \end{array}$$
Cluster Strength: similiarity of F & M to other binomials in female first cluster in prior decade:
  $$\begin{array}{l} \beta_{3}\overset{n}{\underset{i\;=\;1}{\sum }}\left(Sem\text{\_}sim\left(F\&M,\;F_{i}\&M_{i}\right)+Morph\text{\_}sim\left(F\&M,\;F_{i}\&M_{i}\right)\right) \end{array}$$

(9) P(F&M) ~ Accessiblity_F−M_^*^ Competition ^*^ ClusterStrength + decade + (1 + decade | item)

We used a mixed-effect linear model to predict the probability of female-first ordering in 45 gendered binomials, as determined in the first year of each decade 1920–2010 and 2019 (2020 data was not available as of February 2021). Because the decades span a linear period, we treated decade as a numerical fixed effect, and included by-item random intercepts and slopes. As planned, we do not consider the interaction of decade with the other factors, since there is no reason to believe that our three accessibility factors should influence production differently in different decades. We collected percentages of instances for any given year rather than a frequencies, because the size of the Google N-gram corpus differs across years. We then converted percentages to frequencies by multiplying by 1 million. All factors were scaled. Results of the linear mixed model are provided in Table 1.

**Table 1:** Results of fixed effects in the linear mixed model, predicting the P(F&M) order from the relative accessibility of female and male terms, the log-frequency of M&F order, and the cluster strength of related cases as weighted by semantic and morphological similarity.

	**Estimate**	***SE***	***df***	***t*-value**	***Pr*(>|*t*|)**
**P(F&M)** **~** **Competition** * **Accessibility** * **ClusterStrength** **+** **decade** **+** **(1+** **decade | item)**					
**Fixed effects**					
(Intercept)	0.010	0.108	41	0.090	0.929
**Competition**	−0.631	0.063	225	10.025	**0.001**
**Accessibility(F-M)**	0.191	0.064	306	2.967	**0.003**
**ClusterStrength**	0.067	0.033	395	2.052	**0.041**
**Decade**	0.178	0.037	65	4.792	**0.001**
Competition:acc	−0.029	0.043	458	−0.684	0.494
**Competition: cluster**	−0.062	0.027	241	−2.325	**0.021**
acc:cluster	0.058	0.036	248	1.601	0.111
**Competition:acc:cluster**	0.122	0.034	280	3.643	**0.001**
	**Random effects**				
	**Variance**	***SD***	**Correlation**		
Item (Intercept)	0.51863	0.7202			
Decade	0.03758	0.1939	0.28		
Residual	0.04422	0.2103			
		**Model fit**			
	**Marginal**	**Conditional**			
R^2^	0.438	0.959			

*Number of obs: 488, groups: item, 45. Boldface indicates statistical significance*.

As expected, all three proposed accessibility factors as well as decade significantly predicted the probability of female-first order. Since frequencies were calculated based on a single year within each decade, the time series is not continuous. As predicted, higher competition from the male-first order reduces the probability of female-first order (ß_*competition*_= −0.631, *p* < 0.001). The relative accessibility of the female term compared to the male term showed a significant positive effect (ß_*accessibilty*_ = 0.190, *p* < 0.01). There is also an overall effect of decade (ß_*decade*_ = 0.178, *p* < 0.001), indicating that the probability of female-first order has increased over time.

Most relevantly in the current context, results confirm that cluster strength is a significant predictor of female-first order (ß_*cluster*_ = 0.067, *p* < 0.05), beyond the main effect of decade. We find two significant interactions involving cluster strength. The effect of competition (log-frequency of the male-first order) interacts with cluster strength (ß_*competition*:*cluster*_ = −0.062, *p* < 0.05), and there is 3-way interaction between competition, accessibility and cluster strength (ß_*competition*:*acc*:*cluster*_ = 0.122, *p* < 0.001). The 2-way interaction with competition (from male-first order) tells us that the effect of cluster strength is stronger for items that are less entrenched (less frequent) in the competing male-first order. Yet the 3-way interaction essentially tells us that there is a clear positive effect of cluster strength even when frequency of the competitor is high as long as the accessibility of the female term is not particularly low in comparison to the male term.

**Figure 7:**
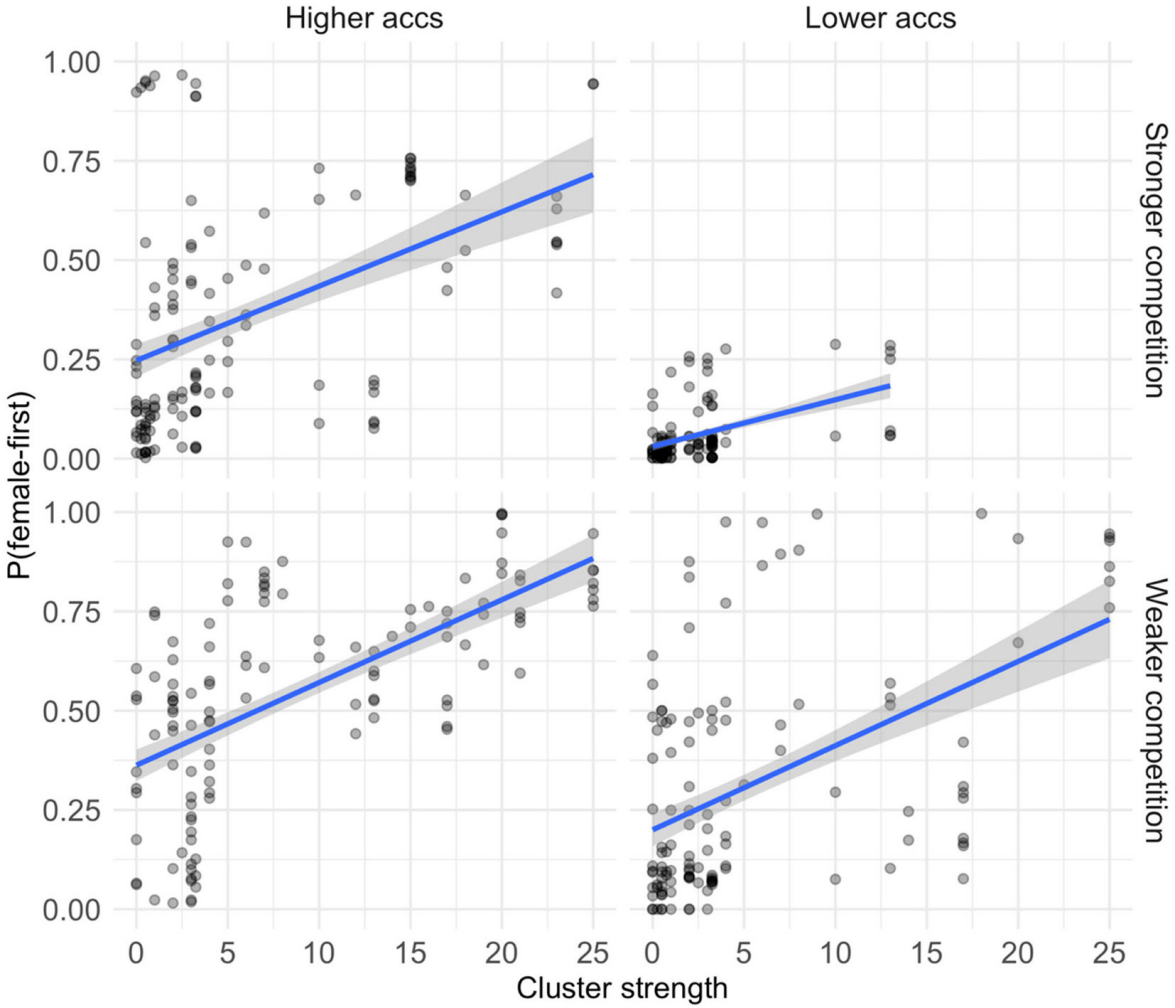
Scatterplots depicting the correlation of cluster strength on the probability of female-first order in the following decade, plotted separately for stronger and weaker competition and for higher and lower relative accessibility of the female term. Data is separated along the median values for both competition (log frequency of male-first order) and relative accessibility. Log-frequency of the binomials to capture the interaction between frequency and cluster strength.

To make sense of these interactions, Figure 7 depicts the correlation between cluster strength and the probability of female-first order, plotted separately for higher and lower values of competition from male-first order, and for the relative accessibility of the female term compared to the male term. Data is separated along the median values for both competition and relative accessibility of the female term. The positive influence of cluster strength on female-first order is evident in the upward slope in all panels: higher cluster strength correlates with higher probability of female-first order. Clearly the top right panel is unlike the others in that it is missing any data with high cluster strength: in our dataset there happened to be no high frequency (strong competition) binomials in which the female term was particularly less accessible than the male term, and which named a pair of roles within a family tree.

## Discussion

All three factors related to accessibility had a significant influence on the ordering of gendered binomials in the current dataset of 45 items. The relative accessibility of the female term compared to the male term can be illustrated most strikingly in the case of the initial cluster—*mother and dad*; *mother and daddy*—which appeared with a female-first preference as early as 1920, influenced by the fact that *mother* was 100x as frequent as *dad or daddy*. Also, as expected from prior work, higher frequency binomials displayed a lower probability of female-first order. Since virtually all gendered binomials except the two just mentioned (and *ladies and gentlemen*) preferred male-first order in 1920, we take this to demonstrate that, the more entrenched binomials were in the male-first order, the easier they were to access, in comparison to the innovative female-first order. Importantly, neither factor on its own can explain the shift in word order, since in the vast majority of cases, the male term was and remained more accessible in absolute terms, and the male-order was originally the preferred order.

Results also show that the probability of female-first order has increased over time. This is consistent with the idea that speakers have come to perceive less of a power differential between males and females, a suggestion foreshadowed in Mollin (2013) as discussed in section Social Influences. The current analysis assigns this change to an independent factor, “decade,” which we acknowledge is rather unmotivated. Recall that we were conservative and simply added a fixed constant to the calculation of accessibility for each male term, which was intended to capture “dominance,” without decreasing it across time. For instance, we could have reduced the dominance constant assigned to male terms by 0.08 at each decade, adding 1.0 to male terms in 1920 and 0.2 today. This would have increased the influence of relative accessibility of the individual terms, while reducing or eliminating the role of “decade.” However, since we had no independent way to verify this, we conservatively kept the dominance constant fixed. In any case, an increase in perceived gender-equality cannot predict the *reversals* in preferred order, since a male-first bias remains operative today; for instance, there is a bias to produce male names first when naming familiar and unfamiliar couples, even with length and closeness controlled for (e.g., Wright et al., 2005; Lohmann and Takada, 2014; Tachihara and Goldberg, 2021).

To account for the historical reversals in preferred word order among roughly a dozen binomials, the factor of particular interest is *cluster strength*. We predicted that an emergent cluster of female-first binomials slowly attracted other binomials that were semantically and/or morphologically similar, and results bear this out. That is, the particular items which reversed their preferred order to become predominantly female-first comprise a semantic cluster: they all name symmetric roles in a larger family tree. The reversals include terms for parents (*ma and pa, mothers and fathers, mama and papa*), terms for grandparents (*grandma and grandpa, grandmother and grandfather*), and *nieces and nephews, aunts and uncles*. As predicted, gendered binomials for items with only weak similarity to the cluster have barely shifted at all, even with relatively low entrenchment in the male-first order (e.g., *king and queen, actor and actress, waiter and waitress*). That is, the current analysis finds that the cluster of cases displaying a female-first preference in one decade (*n*−1) predicts a small but significant probability of other binomials preferring female-first order in the following decade (*n*) in proportion to the semantic and morphological similarity of the binomial to each instance in the prior cluster.

Notably, attraction to a cluster of similar cases is a relatively weak effect. This is expected, as attraction of new cases to a cluster is a slow and uneven process that occurs on the time scale of decades. The attraction of the female-first cluster has not been sufficient to change any cases in which the female term is longer (e.g., *son and daughter, grandson and granddaughter)*, nor have any items shifted which are particularly frequent in the competing male-first order shifted *(husband and wife)*. The fact that cluster strength is a relatively small effect is also clear empirically from the fact that *brothers and sisters* has failed to reverse its preferred order.

**Figure 8:**
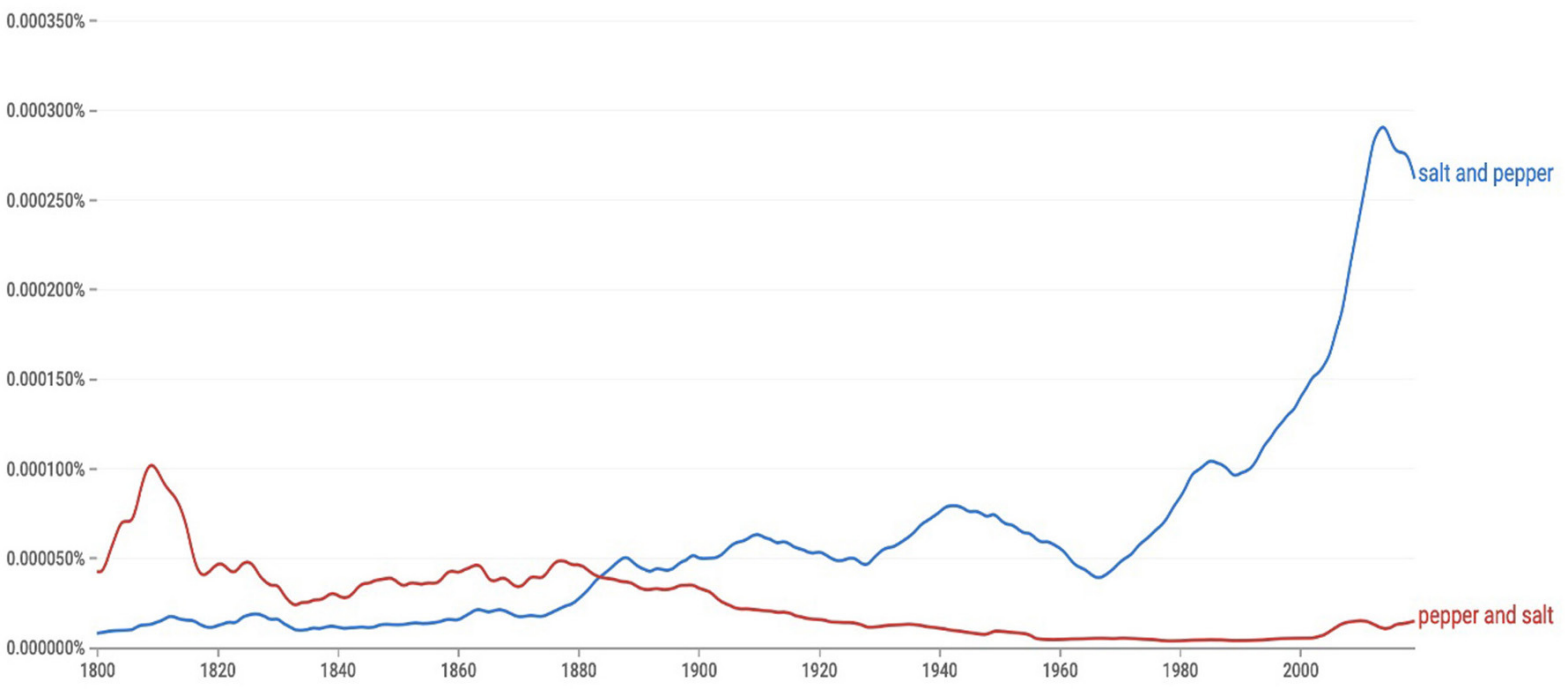
Data from Google Books Ngrams for salt and pepper (blue) and pepper and salt (red).

The role of cluster strength in binomial ordering could be further explored in other cases of historical changes, although as mentioned at the outset, outright shifts in the preferred word order of familiar phrases are quite rare. Candidate reversals among binomials in English include *salt and pepper, math, and science* (Mollin, 2013) and *cheese and biscuits* (Sharon Glass, personal communication, 2/17/21). These cases each came to display the same order preferred today as each phrase gained in frequency, raising the possibility that the apparent preference of the alternative order was affected by small numbers early on (see Figure 8). But to the extent that these cases have reversed their preferred orders, the current account predicts a cluster of related cases may have influenced the changes. For instance, *salt and pepper* may have been affected by a preference for *salt and water/sugar/vinegar*, and *cheese and biscuits*may have been influenced by *cheese and crackers/butter/bread/milk*.

Benor and Levy (2006) observe several “set, open constructions” with a fixed A term and an open B term, here provided in (10)–(12):

(10) nice and <easy/warm/clean/quiet/soft/neat…>
(11) sit and <talk/watch/wait/talk/listen…>
(12) good and <bad/evil/ready/decent/faithful/true…>

On the currentaccount, these three casesconstitute clusterswhich we predict should attract other cases with similarmeanings and/ormorphology^8^.

The current proposal, that accessibility unifies recognized influences on binomial order, finds additional support from the fact that priming and definiteness are recognized to influence binomial order (Benor and Levy, 2006; Morgan, 2016, chapter 3). That is, if one term is primed in the discourse, it can be expected to be temporarily more accessible than it would be otherwise. Relatedly, entities that have previously been evoked in the discourse become temporarily more accessible (Ariel, 1988). The temporary increase in accessibility of one term can lead to the production of that term first, even if the opposite order is otherwise favored. For instance, in example (13) from the COCA corpus (Davies, 2008), *chairs and table* is produced even though the opposite order is favored by roughly 12:1 overall. Presumably, the atypical order in (13) is influenced by the fact that *sat down* primes *chairs*.

(13) She stepped out onto the tiny balcony, a glass of cheap chilled white wine in hand, and **sat down** on an elderly **chairs and table** ensemble that nearly filled the petite balcony (COCA ACAD, 2011, from Davies, 2008, boldface added)

Instances that can be attributed to topicality or relevance are similar. For instance, in general, the order *cars and trucks* is far more prevalent than the reverse order (by roughly 9:1). Yet in example (14), *trucks and cars* is used, presumably because the passage is about noise, which makes trucks more relevant since trucks are noisier than cars (see Tyrkko, 2017 for discussion of intentional stylistic choices).

(14) all night she would hear the **trucks and cars** speeding by (COCA FIC, 2018 from Davies, 2008)

Thus, the recognized role of priming and definiteness in binomial ordering, as well as greater relevance of one term over the other, are consistent with the current claim that accessibility unifies the relevant factors, as these factors straightforwardly increase accessibility.

The current appeal to accessibility implies that the influences related to content, frequency, and length should be consistent across languages, since factors that influence accessibility from memory are presumably universal. That is, we do not expect to find languages that systematically prefer to order the less frequent term in a binomial before the more frequent term, or the less important term before the more important term, or the longer term before the shorter term. Much more cross-linguistic comparisons are needed to investigate this assumption, but early work is consistent. For instance, English and Japanese show remarkably similar semantic influences in binomial ordering despite markedly different word order patterns in other constructions (Lohmann and Takada, 2014; Tachihara and Goldberg, 2021). Polinsky (1997) likewise reported that “importance” influences binomial order in another verb-final language (like Japanese), namely, Tsez, of the Nakh-Daghestanian family spoken in the northeast Caucasus of Russia. That is, she observes that entities deemed to have higher importance in the culture tend to be produced first, e. g., *enij-no kid-no* (“mother and daughter”) rather than # *kid-no enij-no* (“daughter and mother”).

We of course do *not* assume that translations of one binomial into other languages should prefer the same word order: word frequencies and length differences, as well as differences in construal based on cultural differences are expected to vary across languages. Moreover, we have emphasized that learned clusters of cases influence accessibility; this factor entails that word order is influenced by semantically and morphologically related binomials in a given language, in addition to the factors which can be determined by considering any individual binomial on its own.

Prior work on binomial order has not always been explicit about the production process. One exception is Benor and Levy (2006, p. 236), who hypothesize the process as follows.

We assume that every corpus instance of a binomial was generated as follows. First, the speaker/writer determines the individual words constituting the binomial, as well as the context surrounding the binomial. Given the words and context, the speaker/writer then chooses an order in which to produce the words (Benor and Levy, 2006, p. 236).

This proposal presupposes that both words of a binomial are already fully accessed before the speaker chooses one order over the other. Benor and Levy acknowledge that for novel binomials, the speaker may access one term first, although they note that their model does not account for this possibility because it assumes that both A&B terms serve as input to the decision process (Benor and Levy, 2006, p. 238). The current proposal takes a different perspective. By emphasizing the role of cognitive accessibility, we recognize that speakers begin to form an intended message before they fully access all of the required words and constructions needed to express that message (Palmer and Pfordresher, 2003). While the intended message in context provides the most reliable cues for speakers to access appropriate words and constructions, some words and constructions are easier to access than others. When producing a binomial, if one term matches part of the intended message and is fully accessed faster than either the other term or the full binomial, we can expect that term to be uttered first. We take this to account for the influence of the relative accessibility of the individual terms in the current analysis.

## Limitations

The current analysis is based on a relatively small set of 45 binomial terms of English. The dataset is limited because our interest is binomials of common nouns that refer to male and female individuals. A larger scale study that includes more gendered binomials would be worthwhile but is beyond the scope of the current project. Although each of the factors included in the current model has been independently motivated, future work is needed that includes and quantifies additional effects of discourse-pragmatic factors (priming, definiteness, relevance) on accessibility. We anticipate effect sizes to depend on the type of corpus or task used and the particular constructions examined.

We have based our analysis on data from Google Books Ngrams because it is far and away the largest corpus of historical English, with roughly 500 billion words (Michel et al., 2011). At the same time, we acknowledge that the corpus is not ideal, as it is based on books rather than on a representative sample of speech (Pechenick et al., 2015). For instance, we can expect the corpus to underestimate the frequency of informal terms such as *mommy and daddy*. Another drawback in using corpus data to investigate accessibility is that there is no easy way to take age of acquisition into account, although age of acquisition is known to influence accessibility (Ellis and Morrison, 1998). While we rely on the larger Google Ngram data here, we note that the same trends toward reversing the preferred order among gendered binomials is evident in the COHA corpus, a carefully curated corpus of historical American English (Davies, 2010).

We acknowledge several arbitrary choices, including the specific values assigned to degrees of similarity. We note here that we did not guess and check multiple value assignments to find values that worked best, but we were instead conservative and simply chose three fixed points on scales of semantic and morphological similarity at the outset. Remarkably, the current results are readily interpretable and consistent with our hypotheses given these limitations. Future work is needed to investigate the influence of clusters on other types of subregularities in language. It is also necessary to determine whether cluster strength is best calculated in terms of type frequency as we have done here, or whether it is important to instead weight instances in the cluster by their token frequencies. Finally, we registered our analysis (on As.Predicted.org, https://aspredicted.org/see_one.php), but only after data for 14/45 items had been collected, so the analysis cannot count as preregistered.

## Conclusion

We began this paper by presenting a small puzzle that had been overlooked: an entire cluster of familiar binomial terms reversed their preferred ordering from male-first to female-first order over the last century. English speakers in the first half of the twentieth century used to display a robust preference for *uncles and aunts, nephews and nieces, pa and ma*, for instance; today, English speakers are far more likely to produce *aunts and uncles, nieces and nephews, ma and pa*. The shift toward female-first order spread to include a dozen cases, exemplifying a case of what might be called, *constructional diffusion*: a change in one (lexically-filled) construction has led to changes in similar constructions over time, dynamically resulting in an emergent subregularity in English. On the basis of data collected from the Google Books Ngram corpus as elsewhere, we analyzed why and how the change took root and spread.

Ease of retrieval from memory has allowed us to unify a wide range of influences on the accessibility of the parts of binomials (A vs. B) and on the binomial as a unit (A&B vs. B&A). Adopting a usage-based constructionist perspective leads us to treat both words and multi-word units as *constructions*. Therefore, the same factors that influence the accessibility of individual words are predicted to influence the accessibility of constructions, including familiar word combinations. These include frequency, semantics (importance, power, agency, definiteness, closeness to the speaker), priming, interference and neighborhood effects (or *clustering*).

We have suggested that the catalyst which triggered the shift was the arrival on the scene of the phrases, *mother and daddy* and *mother and dad*. These cases preferred female-first order because *mother* was orders of magnitude more frequent than *daddy* or *dad*, and was therefore the more accessible term. As the conventional word order of *mother and daddy* and *mother and dad* became fixed, these binomials slowly began to attract highly similar cases, particularly highly similar cases that were not themselves highly entrenched in the opposite (male-first) order. The hypothesized effect of cluster strength was found to be a significant predictor of the probability of female-first order.

We wish to emphasize that meaning—that is, the intended message in context—provides the most important and reliable cues for accessing constructions: we produce language on the basis of the messages we intend to convey. The analysis of gendered binomial phrases has allowed us to (mostly) control for the effect of the intended message, since the same message can be conveyed regardless of word order (“aunt and uncle”≈ “uncle and aunt”). Moreover, the semantic difference between the individual terms in the gendered binomials considered here (e.g., “aunt” vs. “uncle”) differ primarily along a single semantic dimension (gender). This opportunity to adequately control for meaning has allowed other influences on accessibility to become clear.

The current analysis explicitly unifies the role of the emergent cluster of related cases with factors discovered in previous work. We suggest that all influences on binomial ordering fall under the umbrella of *cognitive accessibility:* ease of retrieval from memory. In particular, the probability of A&B order is predicted by (1) the relative accessibility of the A and B terms individually, (2) competition from B&A order, (3) similarity to and number of related A'&B' cases. The same factors should predict the word order of both familiar and novel binomials in addition to the historical change that we address in the current work.

While we assume that the factors affecting accessibility from memory are shared across all humans, particular constructions are expected to differ in terms of frequency, cultural-based construal, and formal properties such as length and complexity. Moreover, subclusters that are in some way irregular, can emerge as in the current case, because constructions are related to one another in memory. While we acknowledge that studying the relationship between memory retrieval and sentence production on the one hand, and historical word order shifts on the other, is uncommon, we feel the current data argues in favor of doing just that.

## Data Availability Statement

The original contributions presented in the study are included in the article/Supplementary Material, further inquiries can be directed to the corresponding author.

## Author Contributions

AG planned the analyses and drafted the paper. CL implemented the analyses, created the figures, and provided discussion, suggestions, and edits. Both authors contributed to the paper and approved the submitted version.

## Conflict of Interest

The authors declare that the research was conducted in the absence of any commercial or financial relationships that could be construed as a potential conflict of interest.

## Footnotes

^1^It would be interesting to investigate whether the male-first bias has been reduced over time in other cases of gendered binomials beyond the pairs of common nouns considered here. This could be tested by comparing historical trends for conjoined names or by investigating whether older speakers show a stronger tendency to produce male-first binomials compared to younger speakers.

^2^The Google Books N-grams corpus is freely available: https://books.google.com/ngrams.

^3^The binomial, *ladies and gentleman* has also preferred female-first order since at least 1800 (Mollin, 2013). As its female-first preference was evident a century before other binomials began to shift, its influence on other binomials appears to have been minimal, likely because it is used in restricted contexts making it more dissimilar to other cases. Its order appears to be motivated by politeness considerations (Cooper and Ross, 1975; Holmes, 2000; Mollin, 2013), and, we suggest, additionally by accessibility considerations since *gentlemen* is longer than *ladies* and was originally complex (“gentlemen”).

^4^Early examples of *mother and dad* can also be found in texts which presuppose a traditional family structure. For instance, in the following example from 1937, the father's role as the sole breadwinner is presupposed just before *Mother and Dad* is used:

(a) “This is a very good way if it is based fairly on the father's income. …your family should.discuss the matter freely. **Mother and Dad** should decide how much money can be spent for clothing” _COHA−NF_.

^5^Benor and Levy (2006) also find that avoidance of two weak syllables also appears to play a role. We do not include this factor because length has the identical effect in the binomials considered here.

^6^There are hints that neighborhood size is determined by the number of distinct types rather than the number of tokens (De Jong et al., 2003). We leave it to future work to determine whether token frequencies of the instances or the token frequency of the cluster collectively additionally plays a role (see e.g., del Prado Martín et al., 2004).

^7^We also changed the registered analysis to be more conservative. The originally planned analysis was (outcome ~ freq1 ^*^ acc ^*^ cluster + (1|decade) + (1|item)). This analysis finds strong effects for all three accessibilty factors: Competiton (“freq1”), Accessibility and Cluster at *p* < 0.0001. We feel the current analysis is more appropriate, so we report it even though using the registered analysis would have made our current points more forcefully. We also include the log frequency of male-first order as the Competition term as described earlier.

^8^When determining that other binomials are similar in meaning, caution is required. For instance, a person who is *nice and clean* is not necessarily *clean and nice* (but is rather, “pleasantly clean”); someone who is *good and ready* is not necessarily *ready and good* (but is rather, “ready for action”). We expect binomials to be less influenced by the existence of clusters that are dissimilar in meaning, since during production, the strongest accessibility cues depend on the intended message, we expect shared morphology to influence accessibility.

## Appendix

**Table A1:** All 45 gendered binomial terms in the current dataset, listed in male-first order.

*actor and actress*	*grandson and granddaughter*	*pop and mom*
*actors and actresses*	*grandsons and granddaughters*	*son and daughter*
*boy and girl*	*husband and wife*	*sons and daughters*
*boys and girls*	*husbands and wives*	*stepfather and stepmother*
*brother and sister*	*king and queen*	*stepfathers and stepmothers*
*brothers and sisters*	*kings and queens*	*stepson and stepdaughter*
*dad and mom*	*man and wife*	*stepsons and stepdaughters*
*dad and mother*	*man and woman*	*uncle and aunt*
*daddy and mammy*	*men and ladies*	*uncles and aunts*
*daddy and mommy*	*men and women*	*waiter and waitress*
*daddy and mother*	*mr. and mrs*.	*waiters and waitresses*
*father and mother*	*mr. and ms*.	
*fathers and mothers*	*nephew and niece*	
*gentlemen and ladies*	*nephews and nieces*	
*grandfather and grandmother*	*pa and ma*	
*grandfathers and grandmothers*	*papa and mama*	
*grandpa and grandma*	*pappy and mammy*	

*Following Mollin (2013) and Morgan and Levy (2015), we included singular and plural binomial terms for the majority of binomials, since their ordering preferences can differ*.
